# Experimental evaluation of infection, dissemination, and transmission rates for two West Nile virus strains in European *Aedes japonicus* under a fluctuating temperature regime

**DOI:** 10.1007/s00436-018-5886-7

**Published:** 2018-04-28

**Authors:** Eva Veronesi, Anca Paslaru, Cornelia Silaghi, Kurt Tobler, Uros Glavinic, Paul Torgerson, Alexander Mathis

**Affiliations:** 1 National Centre for Vector Entomology, Institute of Parasitology, Vetsuisse Faculty, University of Zürich, Winterthurerstr. 266a, 8057 Zürich, Switzerland; 2 Present Address: Institute of Infectology, Friedrich-Loeffler-Institut, Greifswald Isle of Riems, Greifswald, Germany; 3 Institute of Virology, Vetsuisse Faculty, University of Zürich, Zürich, Switzerland; 4 Section of Epidemiology, Vetsuisse Faculty, University of Zürich, Zürich, Switzerland

**Keywords:** West Nile virus, *Aedes japonicus*, Vector competence, Transmission rate, Virus growth, Fluctuating temperature

## Abstract

West Nile virus (WNV) is continuously spreading in Eastern and Southern Europe. However, the extent of vector competence of *Aedes japonicus* (Theobald, 1901) is controversial. In this work, we elucidated the dynamics of virus growth in this invasive mosquito species. Females of *Ae. japonicus* were reared from eggs collected in the field in Switzerland and fed on bovine blood spiked with two WNV lineage 1 strains (FIN, Italy; NY99, USA). Fully engorged females were incubated for 14 days under a fluctuating temperature regime of 24 ± 7 °C (average 24 °C), 45–90% relative humidity, which is realistic for a Central European mid-summer day. Infection, dissemination, and transmission rates were assessed from individual mosquitoes by analyzing the abdomen, legs and wings, and saliva for the presence of viral RNA. Saliva was also investigated for the presence of infectious virus particles. Overall, 302 females were exposed to WNV strain FIN and 293 to strain NY99. A higher infection rate was observed for NY99 (57.4%) compared to FIN (30.4%) (*p* = 0.003). There was no statistical evidence that the dissemination rate (viral RNA in legs and wings) was different between females infected with FIN (57.1%) compared to NY99 (35.5%) (*p* = 0.16). Viral RNA load of FIN compared to NY99 was significantly higher in the hemocoel (*p* = 0.031) of exposed females but not at other sites (legs and wings, saliva). This is the first study describing the vector competence parameters for two WNV strains in a European population of *Ae. japonicus*. The high dissemination and transmission rates for WNV under a realistic temperature regime in *Ae. japonicus* together with recent findings on its opportunistic feeding behavior (mammals and birds) indicate its potential role in WNV transmission in Central Europe where it is highly abundant.

## Introduction

West Nile virus (WNV) is maintained in an enzootic bird-mosquito cycle, but can cause neuroinvasive disease in humans and horses which act as dead-end hosts (David and Abraham 2016). The virus was first isolated in Africa in 1937 and has spread to all inhabited continents. It was introduced to New York in 1999 and has since become distributed over much of North America (Reisen 2013). In Europe, WNV is continuously spreading in Eastern and Southern Europe, for example, in north-east Italy in 2008, from where it dispersed westwards and southwards (Rizzo et al. 2016). It has established in new areas, such as in eastern Austria, where human infections have been recorded since 2009 (Gossner et al. 2017) (for updated maps of human West Nile fever cases, see www.ecde.europa.eu). Until 2004, all cases in Europe were caused by WNV lineage 1. However, WNV lineage 2 has recently been recorded in Hungary (Bakonyi et al. 2006) being the first record of this lineage outside of Africa. From Hungary, the virus has spread to other countries (Di Sabatino et al. 2014). For example, both strains now circulate in Italy (Barzon et al. 2015).

Mosquitoes are generally considered as the main biological vectors of WNV. The virus has also been detected in other hematophagous arthropods such as ticks (Platonov 2001), but their vector role remains unclear. Further, other modes of transmission between vertebrate hosts (fecal-oral route or preying/scavenging on infected animals) are evident but have so far received comparatively little attention. Direct transmission among humans (blood transfusion, breast feeding, transplacental exposure) has occasionally been described (David and Abraham 2016).

Worldwide, WNV has been isolated from at least 75 mosquito species (Medlock et al. 2005), but *Culex pipiens* (Linnaeus) and *Cx. modestus* (Ficalbi) are implicated as the key vectors in Europe (Hubálek and Halouzka 1999). Transmission of WNV to humans or horses requires mosquitoes with an opportunistic feeding behavior, i.e., taking blood meals from both avian hosts and from mammalian dead-end hosts (“bridge vectors”). *Aedes* (*Hulecoeteomyia*) *japonicus japonicus* (Theobald, 1901) is an invasive mosquito species which has colonized and become highly abundant in areas of Central Europe (Kampen and Werner 2014). Although this mosquito species is known to take blood meals from mammals, it was recently shown to feed to a considerable extent on birds (Schönenberger et al. 2016). Vector competence for WNV was proven in the laboratory for field-collected and laboratory-reared *Ae. japonicus* populations from the USA after feeding on viremic chicken (Sardelis and Turell 2001; Turell et al. 2002a). However, a vector competence study with an *Ae. japonicus* field population from Germany revealed refractoriness to WNV (Huber et al. 2014). All these experiments were done under high and constant temperatures, which are not realistic for the Central European climate. By contrast, a recent preliminary study with field-collected *Ae. japonicus* from Switzerland has shown WNV dissemination (detection of viral RNA in mosquito body parts) and transmission (detection of viral RNA and infectious virus particles in saliva) when pools of orally infected mosquitoes were analyzed. These mosquitoes were kept under a fluctuating temperature regime typical of Central European summers (Wagner et al. 2017).

The aims of the present study were to determine infection, dissemination, and transmission rates of two WNV lineage 1 strains: European strain FIN (isolated in Italy in 2009) and NY99 (isolated in the USA in 1999). The studies were done in field-collected *Ae. japonicus* after incubation under realistic Central European mid-summer conditions.

## Material and methods

### Virus propagation and quantification

Two West Nile virus strains (lineage 1) were used for mosquito oral inoculation: NY99 (NCBI accession number DQ211652 (Borisevich et al. 2006) and FIN (NCBI accession number KF234080 (Lim et al. 2013)). Both strains were kindly provided by S. Becker and J. Schmidt-Chanasit (Bernhard Nocht Institute, Hamburg, Germany) as Vero cell passage 2 (V_2_). The same viral strains were further amplified three times in Vero cells giving V_5_ as final passage history. Briefly, Vero cells were grown in 75-cm^2^ cell culture flasks containing Dulbecco’s Modified Eagle Medium (DMEM) supplemented with 1% antibiotics and fungizone (1000 IU/ml penicillin/streptomycin; 4 μg/ml amphotericin (Gibco, Thermo Fisher Scientific, Reinach, Switzerland) (DMEM complete), and 10% fetal calf serum (FCS, Bioconcept, Allschwil, Switzerland). Vero cells at 75–80% confluence were inoculated with 100 μl of the WNV stocks, incubated for 1 h at 37 °C with 5% CO_2_, and supplemented with additional DMEM (complete with 5% FCS), to a final volume of 30 ml. After incubation for 7 days at 37 °C with 5% CO_2_, 200 μl of the supernatant of this Vero cell passage 1 (V_1_) was inoculated into further 75-cm^2^ flasks containing Vero cells and amplified as described above, generating passage V_2_. The same procedure was repeated to generate V_3_.

Tenfold serial dilutions of V_3_ supernatant were titrated by inoculation of Vero cells in 96-well plates. Briefly, the plates were seeded with 6.2 × 10^5^ cells per well and incubated at 37 °C and 5% CO_2_ for 24–36 h prior to inoculation with virus. For the inoculation, the medium was carefully removed from the wells, 100 μl of each tenfold serial dilution were added to the wells in quadruplicates and the plate was incubated for 1 h at 37 °C and 5% CO_2_. Then, each well was overlaid with 100 μl of DMEM complete supplemented with 5% FCS. Plates were sealed and incubated at 37 °C with 5% CO_2_ for 7 days.

Tissue culture infectious dose (TCID_50/_ml) of both virus strains was calculated (Finney 1978) by considering the cytopathic effect (CPE) at each serial dilution using a microscope. Viral RNA from the V3 supernatant of both virus strains was also extracted using the QIAamp Viral RNA Mini Kit (Qiagen, Hilden, Germany) according to the manufacturer’s instructions, and reverse transcription quantitative polymerase chain reaction (RT-qPCR) was performed.

### RNA amplification

RNA was amplified by RT-qPCR in a CFX96 Touch Real-Time System (Bio-Rad Laboratories, Cressier, Switzerland). The 20 μl reactions included 5 μl RNA, 0.5 μl of each primer and probe (15 and 5 μM, respectively), 3 μl of RNase-free H_2_O, 10 μl of the buffer (2×), and 0.5 μl of iScript advanced reverse transcriptase using the iTaq Universal Probes One-Step Kit (Bio-Rad Laboratories) according to the manufacturer’s instructions. Primers (sense FLI_BRC_F (GGG CCT TCT GGT HGT GTT C), anti-sense FLI_BRC_R (GAT CTT GGC HGT CCA CCT C)), and probe (FLI_BRC_P (CCA CCC AGG AGG TCC TYC GCA A) were kindly supplied by B. Hoffmann (Friedrich-Loeffler-Institut, Greifswald - Insel Riems, Germany). Each reaction was run in duplicate under the following conditions: reverse transcription at 50 °C for 10 min, polymerase activation at 95 °C for 5 min, and 50 cycles of DNA denaturation at 95 °C for 15 s followed by annealing/extension at 56 °C for 30 s. As positive and negative PCR controls, 5 μl of extracted WNV RNA from our virus stock or RNase-free H_2_O, respectively, were used.

### Mosquito rearing

*Aedes japonicus* used in this study originated from the forest adjacent to the Veterinary Faculty of the University of Zürich (47° 23′ 44″ N, 8° 33′ 10″ E, 520 m above sea level). Eggs of *Ae. japonicus* were collected during June and July 2016 and reared according to the protocol described earlier (Balestrino et al. 2014). Briefly, hatched larvae were maintained in plastic trays (approx. 1000 larvae/tray) containing 500 ml of deionized water and liquid larvae food (50% bovine liver powder, 50% tuna meal powder in water), and incubated at 27 °C with 85% relative humidity (RH) under long day (16L:8D) conditions including 1-h dusk and dawn. Approximately 250 pupae, collected from day 5 post-egg immersion, were placed in plastic cups (diameter 7 cm, height 8 cm) with about 150 ml of deionized water inside polyester cubic netting cages (32.5 × 32.5 × 32.5 cm) (Bugdorm 43030F, MegaView Science Co., Ltd., Taichung, Taiwan). The cages containing pupae and emerged adult mosquitoes were provided with cotton soaked with 10% sucrose solution as a carbohydrate source, and kept at the same climatic conditions as above described for the larvae.

### Mosquito inoculation

**Fig. 1:**
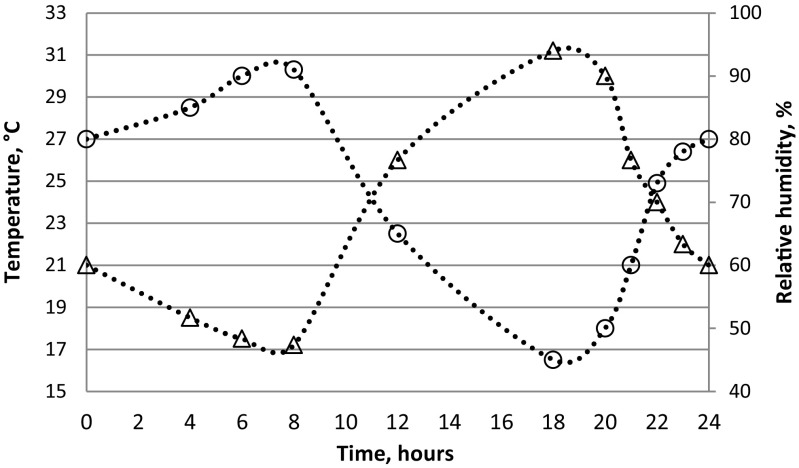
Fluctuating temperature (triangle) and humidity (circle) regimes applied for the incubation of *Ae. japonicus* females orally fed with WNV FIN or NY99

Six to 8-day-old females were deprived of sugar 24 h before exposure to washed heparinized bovine blood, spiked (1:2) with either WNV strain FIN or NY99 cell culture supernatant. Phagostimulant (ATP) at 5 × 10^−3^ M was also added to each blood meal. For oral exposure, virus-spiked blood was transferred to a Hemotek feeder (Hemotek Ltd., Lancashire, UK) covered with a pig intestine membrane. Female mosquitoes were aspirated from the rearing cages and transferred to 500-ml plastic bottles (approx. 50 females per bottle), which had the opening covered with a fine net through which the mosquitoes were exposed to the Hemotek feeder containing the infectious blood. After 30 min of feeding, the mosquitoes were anesthetized by placing the bottles at − 20 °C for few minutes until the mosquitoes were immobilized. Only fully engorged females were collected and transferred (approx. 50 females/box) to a cardboard box cylinder (12-cm diameter and 15-cm length) covered with nets at both sides, with a cotton pad imbibed with 10% sucrose solution on top of the net. Feeding rates (total numbers of fully engorged females among all females exposed to infectious blood) were recorded. The mosquitoes were incubated for 14 days under fluctuating temperature and humidity conditions of 24 ± 7 °C (average 24 °C) and 45–90% relative humidity (reflecting hot spells in northern Switzerland in mid-summer; www.meteoswiss.admin.ch), and a photoperiod of 16L:8D including 1 h of dusk and dawn (Fig. 1). Freshly engorged mosquitoes (two females/virus strain) as “Day 0” infection specimens as well as samples of the infectious inoculum were collected and processed for RT-qPCR providing baseline data for the post-incubated mosquitoes.

### Mosquito dissection and saliva collection

To investigate virus dissemination, legs and wings from live females anesthetized as above were removed and placed individually in 1.5-ml Eppendorf tubes filled with 100 μl of DMEM complete (supplemented with 10% FCS). Females deprived of legs and wings were allocated on a flat surface and their probosces inserted for 30 min into 5-μl glass capillary tubes (Drummond microcaps, Drummond Scientific Company, USA) filled with 10% FCS to collect saliva. The content of the capillary tubes was then expelled into new 1.5-ml Eppendorf tubes filled with 250 μl of DMEM complete. After salivation was terminated, the abdomens were dissected and individually stored in Eppendorf tubes for later investigation of virus infection in the mid-gut. Finally, all samples (legs and wings, abdomens, and saliva) were stored at − 80 °C until further examination.

### Viral detection

Whole insects, abdomens, or legs and wings from each individual female were homogenized using the Tissue Lyser® II instrument (Qiagen, Hilden, Germany). Briefly, one stainless steel bead (3 mm diameter) was added to each tube together with 100 μl of DMEM complete, and the samples were processed with the Tissue Lyser at 25 Hz for 1 min, followed by 5 min centrifugation at 11000*g* and 4 °C. Viral RNA was extracted from each homogenate and from the saliva samples using the viral nucleic acid extraction kit mentioned above, and semi-quantification of viral RNA was performed by RT-qPCR in duplicates.

Saliva samples that resulted positive by RT-qPCR were also tested for infectious virus particles by inoculation of Vero cells in 96-well plates as described above.

### Statistical analysis

The Fisher’s test was used to evaluate differences in the rates of infection, dissemination, and transmission between the two virus strains used, while the Mann-Whitney *U* test was used to assess statistical differences in the amplification efficiency of the two viruses at different stages of infection (mid-gut [analyses of abdomens], hemocoel [legs and wings], and salivary glands [saliva]).

## Results

### Virus quantification of inoculum (Vero cell supernatants)

The final virus titer of V_3_ Vero cell supernatants infected with WNV FIN or NY99, as evidenced by CPE of the tenfold serial dilutions, was 6.25 log_10_ TCID_50/_ml.

### Mosquito inoculation

Overall, 302 and 293 *Ae. japonicus* females were exposed to blood spiked with WNV FIN or NY99, with a final virus titer of 6.0 log_10_ TCID_50/_ml. Day 0 females (two per strain) harbored a mean of 31.1 ± 2.1 Cq/female or 32.4 ± 0.5 Cq/female for FIN or NY99, respectively. Feeding rates were variable for each feeding group (approx. 50 females), ranging between 11 and 44% for FIN strain (overall average 25%) and 10–27% for females exposed to NY99 (average 20%, Table 1). Survival rates 14 days after inoculation were the same (90%) for both strains (Table 1).

**Table 1:** Summary data of experiments with *Aedes japonicus* orally fed with WNV strains NY99 and FIN

Strain	Feeding rate	Survival rate^a^	Infection rate^b^	Dissemination rate^c^	Transmission rate^d^	Transmission efficiency^e^
NY99	60/293 (20%)	54/60 (90%)	31/54 (57.4%)^f^	11/31 (35.5%)^g^	5/11 (45.5%)^g^	5/54 (9.3%)
FIN	77/302 (25%)	69/77 (90%)	21/69 (30.4%)^f^	12/21 (57.1%)^g^	6/12 (50%)^g^	6/69 (8.7%)

^a^14 days after feeding

^b^Proportion of surviving mosquitoes containing viral RNA in their abdomens

^c^Proportion of infected mosquitoes containing viral RNA in legs and wings

^d^Proportion of mosquitoes with disseminated infections that contained viral RNA in saliva

^e^Proportion of surviving mosquitoes with WNV positive saliva

^f^Significantly different (Fisher’s test, *p* = 0.003) between the WNV strains

^g^No statistically significant differences between the WNV strains

#### Infection

**Fig. 2:**
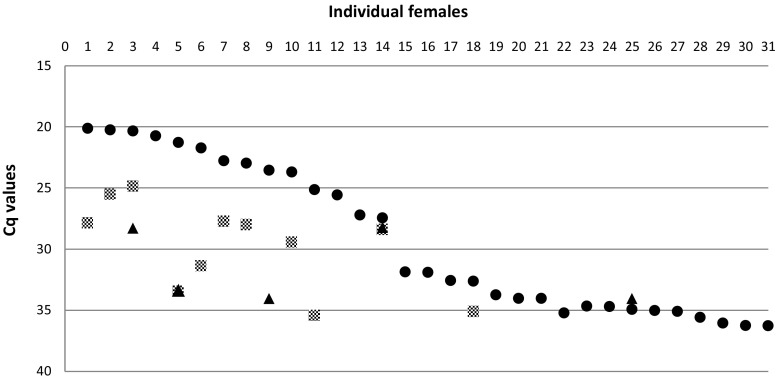
Viral RNA loads (Cq) of females orally fed with WNV NY99. Data are given for infection (data from abdomens, filled circles), dissemination (legs and wings, gray squares), and transmission (saliva, filled triangles). Females’ progression number is reported as from the highest to the lowest viral RNA load in abdomens

**Fig. 3:**
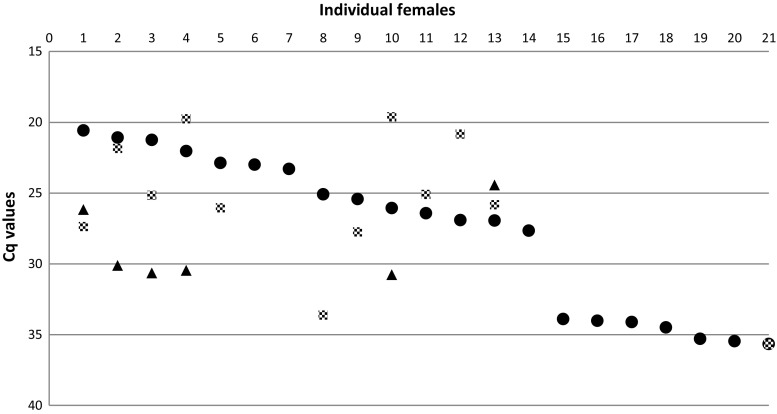
Viral RNA loads (Cq) of females orally fed with WNV FIN. Data are given for infection (data from abdomens, filled circles), dissemination (legs and wings, gray squares), and transmission (saliva, filled triangles). Females’ progression number is reported as from the highest to the lowest viral RNA load in abdomens

The total number of females with viral RNA (≤ 50 Cq) in their abdomen was 45 among those orally fed with FIN (Cq range between 20.6 and 41.9) and 39 with NY99 (Cq range between 20.1 and 46.1). Using a more conservative cutoff (Cq ≤ 36), the percentage of females with viral RNA in their abdomens (infection rate, IR) was significantly higher for mosquitoes infected with WNV NY99 (*n* = 31, 57.4%) compared to those infected with FIN (*n* = 21, 30.4%) (*p* = 0.003) (Table 1). The highest viral RNA loads in abdomen homogenates were very similar among the two strains: 20.6 and 20.1 Cq/abdomen for FIN and NY99, respectively (Figs. 2 and 3). There was no difference with regard to viral amplification efficiencies of the two strains.

#### Dissemination

There was no statistical evidence that the dissemination rate (DR, viral RNA in legs and wings) was different among females infected with FIN (57.1%) and with NY99 (35.5%) (*p* = 0.16) (Table 1). However, the amplification efficiency in the hemocoel of females was higher when infected with FIN strain compared to NY99 (*p* = 0.031). The highest viral RNA load (19.6 Cq) was recorded from homogenates of leg and wings of individual females infected with FIN, while NY99 loads were about five Cq lower, reaching a maximum of 24.9 Cq (Figs. 2 and 3). The range of Cq values recorded among legs and wings homogenates was 19.6–35.7 Cq for females infected with FIN strain and 24.9–35.4 for the ones infected with NY99.

#### Transmission

Quantification of viral RNA extracted from saliva (transmission rates, TR) was calculated only for females that were positive for dissemination (Table 1). Overall, among all the females with dissemination, six had positive saliva for FIN viral RNA (Cq range 24.4–30.8) and five for NY99 viral RNA (Cq range 28.3–34.1). The Fisher’s test did not reveal any significant TR difference among the two WNV strains (FIN 50%, NY99 45.5%). Both strains showed similar levels of viral RNA load (Cq) in females’ saliva, with a peak of 24.4 and 28.3 Cq/saliva for FIN and NY99, respectively. There was no difference with regard to viral amplification of the two strains. Also, there was no difference in transmission efficiency (Table 1) between the proportion of mosquito females with infectious saliva harboring the two WNV strains (FIN 8.7%, NY99 9.3%) (not significant by Fisher’s test).

Saliva samples that resulted positive by RT-qPCR were also positive for virus isolation in cell culture for both FIN (all six samples) and NY99 (two out of five).

Among the processed females that were exposed to WNV FIN, three were negative for viral RNA in both the abdomen and saliva samples despite the detection of viral RNA in legs and wings. These females were not further considered in our analyses.

## Discussion

With our study, we show the rates of infection, dissemination, and transmission in a local population of *Ae. japonicus* after oral exposure to two different strains of WNV lineage 1 (Italian FIN, and American NY99) under fluctuating temperature regime conditions. Transmission efficiency was similar among both strains after 14 days of incubation. Our results contrast to previous reports, where mosquitoes orally infected with WNV strain of lineage 1 (WN02 genotype virus) showed transmission 4 days earlier than those infected with NY99 genotype virus in both *Cx. tarsalis* (Moudy et al. 2007) and *Cx. pipiens* (Ebel et al. 2004). This inferred that this trait (earlier transmission) of WN02 has contributed to the displacement of NY99 (Moudy et al. 2007).

Although the overall transmission efficiency was similar for the two strains described, they exhibited different viral dynamics according to the stage of infection in the vector. The infection rate was significantly lower in the FIN strain (Table 1), but this was compensated by a higher amplification in the hemocoel, reaching 19.6 Cq/female, compared to 24.9 Cq/female of strain NY99 (Figs. 2 and 3). Lower dissemination rates for the WNV NY99 strain were also described in previous work when compared to strain WN02 (Kilpatrick et al. 2008; Moudy et al. 2007; Richards et al. 2014). Although the mechanisms that regulate the vector’s barriers are still unclear, we can hypothesize that the two strains have different affinities to specific cells/organs of the mosquito population tested here. Different virus amplification rates according to different genotypes of WNV have also previously been described for the strain NY99 and lineage WN02. This suggests that WN02 lineage could be more efficient at entering or escaping from mid-gut cells of infected *Cx. tarsalis* (Moudy et al. 2007).

It has been suggested that a threshold of mid-gut infection might be necessary for WNV to establish dissemination or transmission in *Cx. quinquefasciatus* (Girard et al. 2004) and *Cx. tarsalis* (Reisen et al. 2006). Thus, only females with a titer > 3.5 log_10_ PFU in their hemocoel were capable of transmission (Reisen et al. 2006). Previous work has investigated the vector competence of the biting midge species *Culicoides sonorensis* for bluetongue virus using immunohistochemistry (Fu et al. 1999; Jennings and Mellor 1988). This has also demonstrated that a minimum threshold of infectivity within the mid-gut (3.0 log_10_ TCID_50/_ml) was required to ensure a full dissemination throughout the hemocoel, and therefore increase the likelihood of salivary gland infection. However, the degree of viral RNA amplification in the hemocoel of *Ae. japonicus* in our study does not seem to be linearly correlated with a minimum threshold of infection within the gut cells. For instance, the females with values below 20 Cq in their abdomens had disseminations (leg and wings) that varied between 19.8 and 27.4 Cq or between 24.9 and 33.4 Cq for FIN and NY99, respectively (Figs. 2 and 3). Other studies investigating WNV vector competence have demonstrated that feeding mosquitoes directly on viremic animals gave higher infection and transmission rates (Dohm et al. 2002; Turell et al. 2001) than in our study. However, we were able to confirm virus dissemination and transmission with an artificial blood meal spiked with 6.25 log_10_ TCID_50/_ml, which reflects viremia recorded among wild hosts in nature (Turell et al. 2002b).

Vector competence of a Swiss population of *Ae. japonicus* with the same strains of WNV (FIN and NY99) was demonstrated for the first time in a recent pilot study of our group (Wagner et al. 2017). The limitation of the previous work was that vector competence was explored with pools of orally exposed females and not with individuals. Therefore, it could not define the rate of transmission which is crucial to understand the epidemiology of the disease caused by this virus. Moreover, mosquitoes exposed to a blood meal were collected as adult stages in the field, and thus, neither age nor stage of the gonotrophic cycle was known. Consequently, this had putative implications on feeding rates (which indeed were lower than reported in the present study) and the determination of vector competence.

The only other work investigating the vector competence of a European population of *Ae. japonicus* for WNV was carried out in Germany (Huber et al. 2014). This tested field-collected *Ae. japonicus* orally fed with the same WNV virus strain NY99, described in the present study, at a titer of 2 × 10^7^ PFU/ml. Although the sample size was similar to our study (67 engorged females post-incubation), and infectious virus particles among day 0 females (randomly analyzed) were detected on a Vero cell-based assay, none of the examined females were positive for WNV when tested by RT-qPCR. By contrast, around one quarter of the concomitantly investigated *Culex quinquefasciatus* mosquitoes were positive for WNV, and all *Ae. japonicus* specimens tested (*n* = 4) were positive 14 days after oral inoculation with Japanese encephalitis virus. The authors concluded that the local population of *Ae. japonicus*, in contrast to populations from North America (Sardelis and Turell 2001; Turell et al. 2001), was refractory to WNV, and assumed that differences in the genetic background between these populations might be responsible. Indeed, different genotypic signatures were identified among European populations of *Ae. japonicus* (Zielke et al. 2014). Many features influence mosquito vector competence as assessed under laboratory conditions (e.g., mosquito age and nutritional status, incubation temperatures etc.). Therefore, standardization of the methods (e.g., laboratory feeding, Bock et al. 2015) would be highly desirable.

The putative capacity of the Swiss populations of *Ae. japonicus* for transmission of other vector-borne pathogens was demonstrated also for chikungunya (Alphavirus) and dengue (same family as WNV, Flavivirus) viruses with transmission rates of 13.3 and 91%, respectively, after oral exposure (Schaffner et al. 2011).

Investigating the role of local populations of mosquitoes for the transmission of vector-borne pathogens under temperature conditions that reflect realistic field temperatures is very important for assessing the risk of possible epidemics of diseases such as WNV. Indeed, only a few studies have evaluated the temperature effect on WNV dissemination and transmission by incubating orally fed mosquitoes at different constant or fluctuating temperatures (Brustolin et al. 2016; Danforth et al. 2016; Dohm et al. 2002; Vogels et al. 2017) and none of these were for *Ae. japonicus*.

We have demonstrated high transmission rates for WNV among a Swiss population of *Ae. japonicus* under a realistic temperature regime. This, together with recent findings on its opportunistic feeding behavior including mammals and birds (Schönenberger et al. 2016), confirm its potential role in WNV transmission in Europe where the abundance of this species is focally very high.

Further studies should evaluate the kinetics and phenotypic traits of WNV lineage 2 strains in *Ae. japonicus.* WNV of this lineage has shown a higher fatality rate as compared to WNV lineage 1 from previous outbreaks in Romania. Strains of this lineage widely circulate in the eastern part of Europe (Hernandez-Triana et al. 2014) and were detected in birds also in Austria (Wodak et al. 2011) and Italy (Bagnarelli et al. 2011) where *Ae. japonicus* is present.
